# Moral Rationalization Contributes More Strongly to Escalation of Unethical Behavior Among Low Moral Identifiers Than Among High Moral Identifiers

**DOI:** 10.3389/fpsyg.2019.02912

**Published:** 2020-01-08

**Authors:** Laetitia B. Mulder, Eric van Dijk

**Affiliations:** ^1^Department of Human Resource Management and Organizational Behaviour, University of Groningen, Groningen, Netherlands; ^2^Department of Social, Economic and Organizational Psychology, Leiden University, Leiden, Netherlands

**Keywords:** moral rationalization, moral disengagement, moral identity, escalation, behavioral ethics

## Abstract

Occasional acts of immorality are commonplace. One way in which people deal with their own prior immoral acts, is to rationalize why their acts are morally acceptable. It has been argued that such *post hoc* moral rationalizations may contribute to continuation or escalation of immoral behavior. This paper experimentally tests this causal influence of *post hoc* moral argumentation on escalation of immoral behavior and also tests how this depends on people’s level of moral identity. In three experiments we asked participants to generate moral arguments for their past behaviors. The results show that engaging in moral rationalization causes subsequent continuation and escalation of previous immoral behavior, but more so for low moral identifiers than for high moral identifiers.

## Introduction

People occasionally engage in acts of behavior that can be considered as ethically questionable. Most people, for example, will at some point tell a lie, or benefit themselves at the expense of others. Not all these behaviors will be equally severe, and oftentimes it may even be unclear whether behaviors should be seen as unethical. Nevertheless, little sins may become problematic if they are repeated and escalate into worse types of unethical behavior (Ashforth and Anand, 2003; Anand et al., 2005; Martens et al., 2007; Gino and Bazerman, 2009; Zyglidopoulos and Fleming, 2009; Martens et al., 2010; Welsh et al., 2015).

Traditionally, research on ethical behavior has focused on explaining why people engage in unethical acts by focusing on what caused the act; e.g., by asking why people would put their own interests first (e.g., Murphy and Dacin, 2011; Moore et al., 2012), or whether some people are more likely to engage in unethical behaviors than others (e.g., Berry et al., 2007; Tijdink et al., 2016). It should be acknowledged, however, that even good people who care about morality, sometimes engage in unethical behaviors (Bersoff, 1999; Mazar et al., 2008; De Cremer, 2011; Bazerman and Sezer, 2016). As a result, it is equally relevant to understand what follows the act, i.e., how people react once they have engaged in unethical behavior. In the current paper, and in line with literature on moral compensation (Doosje et al., 1998; Wohl et al., 2006; Sachdeva et al., 2009; Jordan et al., 2011; Cornelissen et al., 2013; Mulder and Aquino, 2013) we thus focus on the aftermath of morally questionable behaviors, and address the issue of whether people deal with their transgressions in a compensatory way or in an escalating way. We draw attention to the possibility that people may differ in how they deal with moral transgressions, and that these differences are related to how central morality is to their personal identity because this influences how much people are influenced by *post hoc* moral rationalizations. More specifically, the studies we present in this paper find that engaging in moral rationalization after a moral transgression increases the likelihood of (further) unethical behavior, but more so for those for whom morality is *not* central for their identity.

This paper contributes to the literature on unethical behavior in several ways. First, it contributes to the knowledge about moral identity. Previous research demonstrated that moral identity predicts how people react to their own previous behavior (Mulder and Aquino, 2013), but did not tap into the mechanisms underlying this effect. The current paper sheds light on the mechanism of moral rationalizations that can underlie the influence of moral identity in moral self-management. Secondly, the paper contributes to research on moral rationalizations (Tsang, 2002) and on similar concepts such as moral disengagement (Bandura et al., 1996; Bandura, 1999; Moore, 2008) and neutralizing (Sykes and Matza, 1957; De Bock and Van Kenhove, 2011). The effects of moral rationalizations aimed at justifying one’s *previous* unethical behavior have hardly been explored. Such *post hoc* moral rationalization is especially important to study as it gives insights into the temporal and dynamic dimensions of morality (Shalvi et al., 2015). Thirdly, the paper contributes to literature on the escalation of immoral behavior as it highlights the causal role of moral rationalizations in this. So far this has mainly been highlighted in theory papers (e.g., Ashforth and Anand, 2003; Tenbrunsel and Messick, 2004; Anand et al., 2005; Zyglidopoulos and Fleming, 2009), and in research that studied the role of moral disengagement unethical escalation in a correlational way (Welsh et al., 2015), which still leaves open the question whether engaging in *post hoc* moral rationalizations is actually responsible for unethical behavior escalation. The present paper fills this void and goes a step further by investigating how the role of *post hoc* moral rationalization in escalation depends on moral identity.

## Theoretical Background

Moral identity can be defined as the moral schema people hold about their own moral character (Aquino and Reed, 2002) and refers to the extent to which being moral is central for a person’s sense of self (Blasi, 1984). Moral identity consists of two dimensions: internalization and symbolization (Aquino and Reed, 2002). Internalization refers to the private aspects of the moral self that relate to the traits that are at the core of one’s self concept, whereas symbolization refers to the public aspects of the moral self and reflects traits in actions that are observable by others. Research on moral identity often relies on the internalization dimension of moral identity because it best taps into the extent to which people find morality an important aspect of who they are (e.g., Chowdhury and Fernando, 2014; Joosten et al., 2014; Ding et al., 2016). Also in the present paper, that concerns the influence of private moral argumentation about one’s own previous immoral acts, we refer to the internalization dimension of moral identity when we talk about “moral identity.”

Although moral identity has been shown to positively relate to ethically relevant behaviors (Aquino and Reed, 2002; Reed and Aquino, 2003; Sage et al., 2006; Hertz and Krettenauer, 2015), a high moral identity does not necessarily translate into ethical behavior. As Aquino and Reed pointed out, to translate into ethical behavior, moral identities should be salient in the situation at hand (Aquino et al., 2009). If the situation or decision at hand does not cue one’s moral identity, there is no reason to expect a behavioral effect of moral identity. Thus, it would be inaccurate to say that those who value morality do not engage in morally questionable behaviors. As Gino (2015, p. 107) put it, “immorality is dynamic and malleable,” and individuals may not behave consistently over situations “even when they strongly value morality or when they see being an ethical person as central to their self-concept.” This is especially relevant considering that, oftentimes, we find ourselves in settings in which morality is only mildly at stake. A setting in which the cashier mistakenly hands you back too much change does have a moral ring to it (you might feel that you ‘should’ tell him about this and return the excessive change) but its moral connotation will surely be less strongly evoked than in a setting in which you could steal money from the counter because the cashier does not pay attention. Indeed, we could imagine that many, including those with a high moral identity, may have experienced settings in which they did not correct the cashier’s mistake. In a similar vein, research by Aquino and Becker (2005) indicated that people with a high moral identity were willing to use deceptive strategies in a negotiation if the setting did not cue morality but rather cued financial gains.

The fact that good people can do bad things (Bersoff, 1999; De Cremer, 2011), and that people who value morality may act immorally (Gino, 2015) raises the issue of how people with high versus low moral identities deal with their moral transgressions. Realizing that one just has engaged in morally questionable behavior might stimulate one to do better next time, or even correct and compensate one’s prior behaviors (Zhong and Liljenquist, 2006; Jordan et al., 2011; Mulder and Aquino, 2013; Ding et al., 2016). Alternatively, research has shown that it may be an impetus for escalation (Martens et al., 2007, 2010; Welsh et al., 2015). It has been argued that this path is connected to the moral rationalizations that people use to justify their prior immoral acts (Bandura et al., 1996; Tsang, 2002; Ashforth and Anand, 2003; Zyglidopoulos et al., 2009; Welsh et al., 2015). These can include making up excuses such as “Everybody does it,” “Compared to what I could have done, my act is not so bad” and “Only little harm was done.” Such moral rationalization can be conceived as a form of motivated reasoning (Kunda, 1990) by which people “convince themselves that their behavior does not violate their moral standards” (Tsang, 2002), As such, *post hoc* moral rationalizations may serve to alleviate guilt and self-threat resulting from one’s previous acts and to uphold one’s moral self-image (see also Bandura et al., 1996 and Bandura, 1999 who referred to moral disengagement). Although moral rationalization has been identified as a way that people deal with previous immoral acts (e.g., Shu et al., 2011), it is still unclear to what extent they actually impact further escalation of this behavior. It might be the case that this differs for those with a low moral identity versus those with a high moral identity. This is what we seek to answer in the present paper.

Previous research by Welsh et al. (2015) suggests that moral rationalizations do contribute to the escalation of unethical behavior. However, their research was correlational: both unethical behavior over time and moral rationalizations were measured, and moral rationalizations were tested as a mediator. Assessing mediation can be useful, but does come with some limitations in identifying psychological processes (Spencer et al., 2005). One of the limitations concerns the correlational nature and the fact that the mediation found can be spurious due to an unmeasured third factor that accounts for the relations. For example, in the study of Welsh et al. (2015), the people who were inclined to show unethical behavior (and escalate in this), may also have been the type of persons to be more likely to engage in more moral rationalizations. So their finding does not necessarily imply that engaging in moral rationalization should be seen as the cause of the escalation. Then, the question remains what the mere effects of moral rationalizations are. And, more specifically, what the effects of moral rationalizations are for people with different kinds of general inclinations to show unethical behavior (e.g., high and low moral identifiers).

So how can the influence of moral rationalization on the escalation of unethical behavior depend on moral identity? Although it might seem that *post hoc* moralization is a more important strategy for people with a high moral identity (because they suffer the highest moral self-threat after immoral behavior), we argue that moral rationalizations would have little impact on the escalation of unethical behavior among those with a high moral identity. Our reasoning for this is that rationalizations may only work to the extent that they are successful in convincing oneself of their validity and thus in reducing guilt. In other words, generating moral rationalizations might be of little or no use for those who find them unconvincing. It seems plausible that this will apply mostly to those with a high moral identity. As high moral identifiers are more likely to recognize violations of their moral and social values (c.f., Skarlicki et al., 2008), they might also be more likely to be conscious of the immoral nature of their own past acts. So, if morality is important to you, you might be less convinced by an argument that keeping excessive change would be OK. If so, it could keep those with a high moral identity from treading the escalation path.

Some first evidence that moral identity may modulate how people deal with past transgressions was provided by Mulder and Aquino (2013). In a series of studies they showed that those with a high moral identity were more likely than those scoring low on moral identity to compensate past unethical acts (e.g., lying in a deception game; benefiting oneself at the expense of others) by subsequent ethical acts (being honest; donating to charity). Note, however, that these studies did not investigate (or assess) moral rationalizations, making it unclear whether the actions of those high in moral identity were due to a lower engagement in moral rationalizations as compared to those with a low moral identity. Another relevant study was conducted by Aquino et al. (2007), who investigated to what extent American students reported negative emotions in response to the norm violating behaviors US soldiers in the Abu Graibh prison. Measuring the participants’ agreement with justifications for the soldiers’ behavior (e.g., “Taking embarrassing photos of Iraqi prisoners is no big deal when you consider the harm Iraqis have brought to so many people”), they found that those who agreed more with such justifications felt less negative about the transgressions. Importantly, this correlation was observed among people with a low moral identity but not among those with a high moral identity. This pattern fits the current proposition that moral rationalizations may be more effective for those with a low moral identity than for those with a high moral identity. Note, however, that this study was correlational. Moreover, the dependent variable in the Aquino and colleagues study were judgments about unethical acts performed by others, rather than one’s own (further) engagement in unethical behavior. Consequently, the study touched on the moral rationalization process, but did not directly test its influence on escalatory unethical behavior.

## Approach

We aimed to shed more light on the process of moral argumentation after a past unethical act, and test whether the process would be different for high moral identifiers than for low moral identifiers. To increase the understanding of how moral arguments affect behavior, and to establish the causal path, we therefore asked participants to provide moral arguments for their past behaviors, and we studied its impact on future behavior. While the main theoretical focus was on whether and how moral rationalizations might affect future behavior, we also included a condition in which participants were asked to provide moral objections (i.e., reason why unethical behavior was wrong) rather than rationalizations. We included this to control for the possibility that the findings we obtained might be explained by a mere increase in salience of the morality concept. If it is moral rationalization that drives future behavior rather than morality salience, effects should primarily emerge when participants are asked to engage in rationalization. We present three studies in which we assessed the participants’ moral identities and had them generate moral rationalizations (depicting the immoral behaviors as being acceptable) versus moral objections (depicting the immoral behaviors as being inacceptable). To ensure that effects were uniquely connected to the aftermath of immoral behavior, we also manipulated whether prior behavior pertained to ethical vs. unethical behavior. After all, if we would find that moral rationalizations only evoke unethical behavior after previously having shown unethical behavior (and not after previously having shown ethical behavior), then this suggests that moral rationalizations contribute to the *escalation* of one’s prior unethical behavior and not so much as an independent instigator of unethical behavior in itself. In agreement with past research we expected moral rationalization to increase subsequent unethical behavior, but primarily after having performed immoral behavior. This setup allowed us to test whether moral rationalization would impact high moral identifiers more or less than low moral identifiers.

For all experiments, we have reported all measures, conditions, data exclusions, and mention how we determined the sample sizes. The data of all studies and supplementary material are publicly available at https://osf.io/rkgcs/files/.

## Study 1

To provide a first test of our (three-way) interaction hypothesis, we assessed our participants’ moral identity before presenting them a scenario depicting a setting in which they had or had not engaged in morally questionable behavior. We opted for a mild transgression, to prevent that the participants would not picture themselves showing the behavior in the first place. After reading the scenario, participants were either requested to generate moral rationalizations (i.e., write why their behavior had not been problematic), or generate moral objections (i.e., write why their behavior had been problematic).

### Method

#### Participants and Design

A power analysis (using G^∗^Power 3.1, *F*-test) indicated that – to obtain significant medium effects (*f* = 0.25) with a statistical power of 0.80 per effect and an alpha of 0.05 – we needed at least 128 participants. In the context of a research method class of a European University, eight students aimed to recruit 50 participants each (in their social environments, in public places, and in various organizations they had access to). This led to 373 participants (170 males, 197 females, 6 unknown; *M*_age_ = 32.4 years, *SD*_age_ = 13.56). Participants had a wide variety of jobs and educational background (32% university education, 27% higher vocational education, 21% lower vocational education, and 13% high school). They were randomly assigned to one of the conditions in the 2 (prior behavior: unethical vs. ethical) × 2 (moral argumentation: moral rationalization vs. moral objection) factorial design. Moral identity was a (measured) continuous independent variable.

#### Procedure

Participants first filled in the internalization subscale of Aquino and Reed’s (2002) moral identity measure and, after that, another personality scale that was unrelated to this study. This internalization subscale of moral identity taps into the degree to which moral traits are central to the self-concept and has been used in several studies of moral functioning (Aquino and Reed, 2002; Reynolds and Ceranic, 2007; Detert et al., 2008; Aquino et al., 2009). The measure presents respondents with nine characteristics that might describe a person (i.e., caring, compassionate, fair, friendly, generous, helpful, hardworking, honest, and kind), and then asks them to visualize “the kind of person who has these characteristics [and] imagine how that person would think, feel, and act.” After this, on a seven-point Likert response scale (1 = strongly disagree, 7 = strongly agree) participants indicated their agreement with five items: (1) “It would make me feel good to be a person who has these characteristics,” (2) “Being someone who has these characteristics is an important part of who I am,” (3) “I would be ashamed to be a person who had these characteristics (reverse scored),” (4) “Having these characteristics is not really important to me (reverse scored),” and (5) “I strongly desire to have these characteristics” for each of the items. The items were averaged to determine the moral identity score for each participant (α = 0.73).

All participants subsequently read a scenario describing an international company in which employees often go on business trips abroad for which travel costs, including meals, are reimbursed. To offer some information that could later on be used for generating moral rationalizations, the company was described as being a bit cheap on salary and bonuses and participants were asked to imagine feeling slightly underpaid. The scenario described a business trip in China, in which they had dined with three others. The bill was in Chinese and therefore hard to interpret. They could only figure out the total amount, which in terms of euros amounted to €40. They all had split the bill, each of them only paid €10. In the *previous ethical behavior condition*, participants imagined that they subsequently reimbursed the €10. In the *previous unethical behavior condition*, participants imagined that they had reimbursed the total amount of €40.

Then, the moral argumentation manipulation was induced by an argumentation assignment. In the *moral rationalization condition* participants were asked, with the first reimbursement scenario in mind, to write a plea in which they argued why it would be okay (either according to themselves or to others) to reimburse the whole bill. They were asked to write down these reasons as convincingly as they could. It was stressed that it did not matter whether they personally agreed to this reasoning and that, even if they might not agree, the task was to write down good reasons why it would be okay. In the *moral objection* condition they had the same assignment, except that they were asked to write down reasons why it was not okay to reimburse the whole bill.

After this, all participants were presented a second scenario, this time concerning a business trip in Jordan. They imagined to have dined with three partners, split the bill and paid their share of the bill.^1^ The bill was written in Arabic so that only the total amount was clear and that it could also be interpreted as an already split-up bill for one person. Then, behavioral intentions regarding their reimbursements were assessed by asking participants to what extent they were inclined to reimburse the full amount without mentioning that the bill was for four persons (1 = absolutely not, 7 = absolutely).^2^

Finally, it was checked whether participants had filled in the survey in the correct order and they were thanked for their participation.

### Results

Participants who did not fill in this argumentation task (6), said to have filled it in afterward (2), or clearly argued contrary to what they were assigned to do (15), were excluded from our analyses. Moral identity was standardized, and for the regression analyses cross products were calculated for the interaction terms. Prior behavior and moral argumentation were effect coded (−1 vs. 1).

#### Reimbursements

**FIGURE 1:**
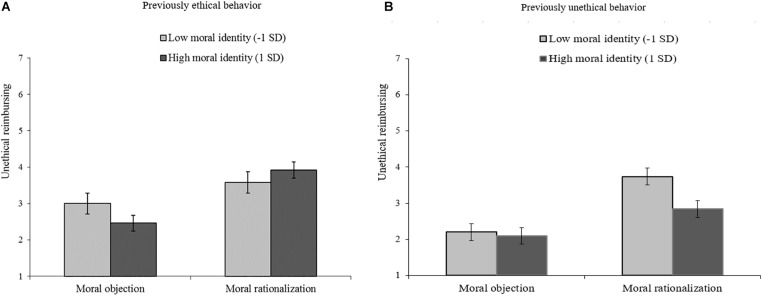
Influence of moral argumentation and moral identity on unethical reimbursing for the previously ethical behavior condition (panel **A** on the left) and for the previously unethical behavior condition (panel **B** on the right), Study 1.

Regression analyses were used to test how moral identity, in combination with our manipulations, affected reimbursement intentions. We tested our hypothesis in three regression steps, shown in Table 1. First, unethical reimbursement behavior was regressed on the moral argumentation, prior behavior, and moral identity. This model was significant *F*(3,347) = 9.76, *p* < 0.001, *R*^2^ = 0.08) and yielded main effects for moral argumentation (*B* = 0.55, *p* < 0.001) and prior behavior (*B* = 0.27, *p* = 0.02). Second, the two-way interactions were included, which did not significantly change the explained variance. The main effect of moral argumentation and the absence of an interaction between moral argumentation and previous behavior suggests that moral argumentation in itself affects the behavior that follows it, but that moral argumentation, on the whole, does not contribute to escalation of previous unethical behavior. When the hypothesized three-way interaction was included in the third step, this changed the model in a marginal significant way, Δ*F*(1,343) = 3.07, *p* = 0.08, *R*^2^ = 0.09, Δ*R*^2^ = 0.01, and the Moral Argumentation × Prior behavior × Moral Identity interaction was marginally significant (*B* = 0.21, *p* = 0.08). This interaction is plotted in Figures 1A,B.

**TABLE 1:** Results of Hierarchical Regression on unethical reimbursing as a function of moral argumentation, previous behavior and moral identity (Study 1).

	**Step 1**	**Step 2**	**Step 3**
	***B***	***B***	***B***
Moral rationalization (yes)	0.55^∗∗^	0.55^∗∗^	0.54^∗∗^
Previous behavior (moral)	0.27^∗^	0.27^∗^	0.26^∗^
Moral Identity	–0.13	–0.13	–0.15
Moral rationalization × Previous behavior		–0.02	–0.03
Moral rationalization × Moral Identity		0.02	0.01
Moral Identity × Previous behavior		0.09	0.10
Moral rationalization × Moral Identity × Previous behavior			0.21^+^
*R*2	0.08^∗^	0.08^∗^	0.09^∗^
Δ*R*^2^		0.00	0.01^+^

*^+^*p* < 0.10; ^∗^*p* < 0.05; ^∗∗^*p* < 0.01.*

Follow-up analyses of this interaction showed that when prior behavior had been unethical, low moral identifiers who had generated moral rationalizations were more inclined to engage in unethical reimbursement than those who had generated moral objections (*B* = 0.76, *p* < 0.001). For high identifiers this relation was not significant (*B* = 0.38, *p* = 0.10). These patterns suggest that, after prior unethical behavior, moral rationalization increases subsequent unethical behavior more strongly for low moral identifiers than for high moral identifiers. We also reasoned that after having shown ethical behavior, the subsequent generation of moral rationalizations (versus moral objections) should have less impact, as no prior behavior needed to be justified. In agreement with this, we observed a non-significant effect among those with a low moral identity (*B* = 0.30, *p* = 0.23). In contrast to this notion, however, we did observe a significant effect among those with a high moral identity (*B* = 0.73, *p* = 0.001). We return to this observation in the Discussion.^3^

### Discussion

The results suggest that, overall, engaging in moral rationalizations does not induce continuance of prior unethical behavior *per se*, but that it does so more strongly for low moral identifiers. The three-way interaction supports a motivational account, showing that generating moral rationalizations only affects low moral identifiers’ unethical behavior if prior to this they engaged in unethical behavior. Moral rationalizations thus did not evoke unethical behavior for those who previously had chosen the moral path.

The finding that generating moral rationalizations after prior unethical behavior promotes further unethical behavior more for those with a low moral identity than for those with a high moral identity, suggests that having a high moral identity may reduce the likelihood to further tread the path of escalation or continuation. Our interpretation is that for those with a high moral identity, these self-generated moral rationalizations are less likely to serve as a viable excuse for further unethicality. This favors the explanation that low moral identifiers are more likely than high moral identifiers to conclude from their own moral rationalizations that the behavior is morally acceptable, and feel less guilty about their previous acts.

In our introduction, we reasoned that the type of generated arguments (moral rationalizations vs. moral objection) would have little or no effect if one would previously have behaved ethically. While this was true for those with a low moral identity, we found that high moral identifiers who had previously shown the moral behavior (i.e., only reimbursed their own share of the bill) were affected by the generation of arguments. They were subsequently less likely to unethically reimburse the total amount after having generated reasons as to why it would not be OK to reimburse the entire bill than after having generated reasons as to why that would be OK. A plausible *post hoc* explanation for this finding is that after having just behaved ethically, generating moral objections to immoral behavior may affirm high moral identifiers in the importance they ascribe to being ethical, making them (even) less susceptible to make future unethical reimbursements. Study 2 allowed us to test whether this unanticipated effect would replicate.

## Study 2

Study 2 had the same design as Study 1. Its first aim was to replicate the finding in Study 1 that, after a prior immoral act, moral rationalization (as compared to moral objection) would increase subsequent unethical behavior, but more so for low moral identifiers than for high moral identifiers. Second, to contribute to external validity of the results, Study 2 focused on a different type of unethical behavior. Reasoning that the type of unethical behavior in Study 1 (reimbursing costs of dinners) may not have been a standard that our participants had often encountered in their lives, we used a setting that would probably be more familiar (i.e., being given back too much change in a sales interaction)^4^. Another improvement was that, in Study 2, we used a long time lag between the measurement of moral identity and the actual study. This is to rule out that the results with regard to moral identity can be attributed to the temporal salience of morality due to the moral identity questionnaire.

### Method

#### Participants and Design

The same power analysis as in Study 1, that indicated a desired minimum of 128 participants, applied to Study 2. We aimed for 160 participants (psychology undergraduates who participated for course credits) and ended up with recruiting 150 (39 males; *M*_age_ = 19.35, *SD*_age_ = 1.90). They were randomly assigned to one of the conditions in the 2 (prior behavior: unethical versus ethical) × 2 (moral rationalization: moral rationalization vs. moral objection) factorial design. Moral identity was again measured as continuous independent variable.

#### Procedure

In the beginning of the year, participants filled in a test battery of personality questions, including moral identity. Moral identity was measured in the same way as in Study 1 (α = 0.79). About a month later, they were re-invited to the laboratory and filled in a paper-and-pencil survey that was called an “Argumentation Study.” First, they were explained the global idea of the argumentation task they were about to engage in. Then, they read the scenario.

Participants were asked to imagine that they were doing groceries in the supermarket and, because they were a bit short on money, they restricted themselves to cheap products. Next, they imagined that, after 10 min in the queue, they checked out, but that the cashier, being absent-minded, gave back too much change, namely €5 too much. In the *prior unethical behavior condition* they were asked to imagine that they did not mention the mistake and walked away with €5 too much. In the *prior ethical behavior condition* they were asked to imagine that they said “This is €5 too much” and returned the money to the cashier.

Subsequently, participants engaged in the argumentation task. As in Study 1, participants in the *moral rationalization condition* wrote a plea in which they rationalized that keeping the money was okay, and participants in the moral objection condition argued that keeping the money was not okay. Only now, this was done by means of five statements of which participants were requested to, irrespective or their own opinion, argue in favor of or against. Again, it was stressed that it did not matter whether they agreed with these arguments, but that the only important thing was that they argued as convincingly as possible. The five statements were: (1) It is unprofessional of the cashier to be so absent-minded, (2) Keeping the €5 is disadvantageous for the cashier, (3) Keeping the €5 is, in fact, stealing, (4) It is not my responsibility that the supermarket loses €5, (5) Most others would keep the money. So, statements 1, 4, and 5 were moral rationalizations and statements 2 and 3 were moral objections. Behind each statement it was clearly printed whether they should argue in favor or against the statement, and enough space available to write down a short story. In the moral rationalization condition, participants were asked to argue in favor of statements 1, 4, and 5, and against statements 2 and 3. In the moral objections condition this was the other way around: participants were asked to argue in favor of statements 2 and 3 and against statements 1, 4, and 5.

Then, ethical behavior was measured by presenting them with another scenario. In this scenario, they were asked to imagine that they were at the market and were paying the greengrocer, took the change and walked on. About 20 m further, while they were putting the change in their wallets, they noticed that the greengrocer had given them back too much money (€10, which is $12). Unethical behavior was measured by the question “What do you do? Do you go back to correct the mistake or do you walk on and keep the €10?” This was measured on a four-point Likert scale (1 = I absolutely go back to correct the mistake, 4 = I absolutely walk on and keep the €10). So, higher values reflected stronger unethical behavior intentions^5^.

Finally, participants were asked whether they had ever experienced a setting in which they had received too much change (see Footnote 6), after which they were thanked for their participation.

### Results

We excluded two participants from further data analyses. One participant for not filling in the argumentation task, and one for writing an argument for only one statement, that was also in the opposite direction of what he/she was assigned to do. Moral identity was standardized, and for the regression analyses cross products were calculated for the interaction terms. Prior behavior and moral argumentation were effect coded (−1 vs. 1).

#### Unethical Behavior

**FIGURE 2:**
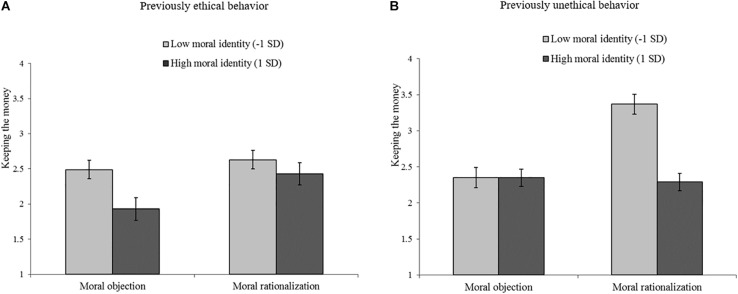
Influence of moral argumentation and moral identity on unethical behavior (keeping the money) for the previously ethical behavior condition (panel **A** on the left) and for the previously unethical behavior condition (panel **B** on the right), Study 2.

The hypothesis was tested in three regression steps (see also Table 2). First, unethical behavior was regressed on the moral argumentation, on the prior behavior, and on moral identity. This model was significant *F*(3,141) = 4.12, *p* = 0.008, *R*^2^ = 0.08) with main effects for moral argumentation (*B* = 0.17, *p* = 0.01) and moral identity (*B* = −0.17, *p* = 0.01). Second, the two-way interactions were included, which did not significantly change the explained variance. Third, the hypothesized three-way interaction was included. This changed the model significantly, Δ*F*(1,137) = 6.93, *p* = 0.009, *R*^2^ = 0.14, Δ*R*^2^ = 0.04, and the Moral Argumentation × Prior Behavior × Moral Identity interaction was significant (*B* = 0.18, *p* = 0.009). This interaction is plotted in Figures 2A,B.

**TABLE 2:** Results of Hierarchical Regression on unethical behavior (keeping the money) as a function of moral argumentation, previous behavior and moral identity (Study 2).

	**Step 1**	**Step 2**	**Step 3**
	***B***	***B***	***B***
Moral rationalization (yes)	0.17^∗^	0.16^∗^	0.20^∗∗^
Previous behavior (moral)	–0.10	–0.09	−0.11^+^
Moral Identity	−0.17^∗^	–0.19^∗∗^	–0.23^∗∗^
Moral argumentation × Previous behavior		–0.04	–0.04
Moral argumentation × Moral Identity		–0.08	–0.09
Moral Identity × Previous behavior		–0.01	0.04
Moral argumentation × Moral Identity × Previous behavior			0.18^∗∗^
*R*^2^	0.08^∗^	0.09^∗^	0.14^∗^
Δ*R*^2^		0.01	0.04^∗∗^

*^∗^*p* < 0.05; ^∗∗^*p* < 0.01; ^+^*p* < 0.10.*

Follow-up analyses of this interaction showed that, when prior behavior was immoral, moral rationalization led to more unethical behavior than moral objection among those with a low moral identity (*B* = 0.51, *p* < 0.001). Moral argumentation did not significantly affect those with a high moral identifiers (*B* = −0.04, *p* = 0.72). When prior behavior was moral, moral argumentation did not increase affect reimbursing among high moral identifiers (*B* = 0.25, *p* = 0.14), nor among low moral identifiers (*B* = 0.07, *p* = 0.60). This supported the hypothesis that, when prior behavior is unethical, moral rationalization increases subsequent unethical behavior more so for low moral identifiers than for high moral identifiers and that this is not the case when prior behavior is ethical^6^.

### Discussion

Study 2 replicated the results of Study 1 for a different kind of immoral behavior. Again, the results show that after having behaved unethically, engaging in moral rationalizations promotes unethical behavior more among low moral identifiers than among high moral identifiers. Now, no strong indication was found that generating moral objections has positive effects for high moral identifiers who have behaved ethically.

## Study 3

Studies 1 and 2 show that moral rationalizations may instigate future unethical behavior, especially if one’s prior behavior was unethical, and for those with a low moral identity. We were able to demonstrate this by using scenarios that allowed us to vary the prior behavior (ethical versus unethical). We, of course, realize that a limitation of this method is that the prior behavior was not self-chosen, and that decisions were hypothetical. We designed Study 3 to study whether the observed relations also generalize to actual unethical behavior. In this study, we first assessed participants’ actual degree of unethicality in a first setting, then had them generate moral arguments, after which we assessed their degree of unethicality in a second setting. This setup enabled us to compare prior and subsequent unethical behavior.

This setting also allowed us to address another issue. Escalation can take the form of engaging in new unethical acts, but also in increasing the level of unethicality in such acts. Studies 1 and 2 could not distinguish between both forms since the participants could not freely select the magnitude of their (new) unethicality. To distinguish between escalation by engaging in a new acts at a similar level of intensity and escalation by engaging in new acts at a higher level of unethicality, we used a setup that allowed participants to deceive another person, but also to select the extent to which they would deceive the other. This setup allowed us to test whether escalation would take the form of continuing in (new) unethical acts at a similar level of unethicality, or at an increased level. Moreover, it allowed us to test whether these forms would be observed more among low moral identifiers than among high moral identifiers.

### Method

#### Participants and Design

Study 3 did not manipulate prior behavior and thus only had two conditions (moral argumentation: moral rationalization vs. moral objection) to which participants were randomly assigned. Moral identity was a (measured) continuous independent variable. We aimed for 120 business students who participated for course credits or money, and ended up with recruiting 109 (59 males; *M*_age_ = 19.73, *SD*_age_ = 2.04).

#### Procedure

Participants were invited into the laboratory and were guided into a cubicle in which the computer experiment took place. First, they filled in a battery of personality questions, among which moral identity scale (α = 0.67).

Then, the participants presented a setting that was modeled after Gneezy’s (2005) cheating game. Participants were informed that they would be paired to another research participant in a task that would involve the distribution of money. They were told that, at the end of the entire study, one of the pairs would be randomly selected and that its members would obtain the monetary outcomes of the allocation they had made. All participants learned that in their pair, they were “player 1” and their partner was “player 2.” Subsequently, the rules of the monetary allocation task were explained. It was explained that player 2 would be presented with 10 options, that each represented a certain distribution of money. Player 2’s task was to pick one of the options, but was not informed what distribution each option represented. The participants did know the distributions for the options, and – knowing this – had to advise player 2.

Being informed of the distribution, the participants learned as player 1 that the 10 options (labeled option A to option J; see Appendix) represented distributions of 50 euros, that were increasingly more profitable for themselves, but increasingly less profitable for player 2. Option A was thus the most advantageous option for player 2 (Allocating 50 euros to player 2 and 0 to player 1), and option J was the most disadvantageous for player 2 (allocating 0 euros to player 2 and 50 euros to player 1). Participants could then only inform player 2 which option was most advantageous for player 2. Since player 2 was unaware of the actual distribution, this allowed participants to deceive their partner. Participants could choose from 10 messages that corresponded to the 10 options A–J. For example, message 3 was “Option C will earn you the most.” While an honest message would require participants to send message 1 and thus inform player 2 that A was the most advantageous option, sending messages 2–10 (referring player 2 to options B–J) would be increasingly deceptive, and increasingly in the own advantage. It was stressed that player 2 would never find out about the distributions attached to the different options. The only thing (s)he would know was – if the bonus would be awarded – how much (s)he got awarded him/herself.

After participants had made their decision about which message to send, they engaged in an argumentation task in which moral rationalization was manipulated. Moral argumentation was manipulated as in Study 1, this time pertaining the act of sending an untruthful message.

Then, they were presented with the same decision for the second time (it was made clear that this would be the last time). It was stressed that the person to whom they were coupled this second time, was a different person than the one they were coupled with the first time. The main characteristics of the decision situation were repeated, and they were again asked to choose between the 10 messages. Finally, participants were debriefed and, 1 week later, one participant was randomly selected and awarded a bonus according to his/her decision^7^.

### Results

#### Chosen Messages

As a first identification of the main messages the participants sent, we explored the frequency data. These data showed that there were clearly four messages that were chosen most frequently sent to player 2 (covering 85% of all messages sent in round 1, and 87% in round 2). One of the four mostly used messages was to truthfully conveyed that option A was the most advantageous for player 2. This message was chosen by 15% of the participants in round 1 and 19% in round 2. All other participants sent a deceptive message. A high percentage of the participants (40% in round one and 35% in round two), informed player 2 that it would be most advantageous to select option F; the option that yielded an almost equal distribution, but slightly in favor of the participant. Another part of the participants (20% in round one and 22% in round two) informed player 2 that option E was most advantageous; the option that also was close to an equal distribution, but slightly in favor of player 2. The most extreme form of deception, identifying the option that was most advantageous to the participant as the option that would be most advantageous to player 1 was used by 10% of the participants in round 1 and 11% in round 2).

With regard to the difference between round 1 and 2, a paired sample *t*-test showed that respondents told slightly less extreme lies in round 2 (*M* = 5.67, *SD* = 2.37) than in round 1 (*M* = 5.43, *SD* = 2.68), *t*(108) = 1.75, *p* = 0.08. Although this was a marginal effect, it suggests that, overall, respondents were more inclined to de-escalate than to escalate in their deceptive behavior. There was no correlation between moral identity and deception in the first round (*r* = 0.14, *p* = 0.13) or in the second round (*r* = 0.001, *p* = 0.99). So there was no indication that high moral identifiers were less likely to send a deceptive message than low moral identifiers, which supports the notion that “good people” (i.e., those who strongly value morality) can actually “do bad things” (Bersoff, 1999; De Cremer, 2011; Gino, 2015).

#### Escalation

As Studies 1 and 2 indicated, moral rationalizations primarily promote subsequent unethical behavior if the prior behavior was unethical as well. Accordingly, we first analyzed the behavior of those who had not deceived player 2 in round 1. This exploration indeed indicated that all of these participants also decided to truthfully inform their opponent in round 2.

To identify possible escalation (or de-escalation) we therefore proceeded with analyzing only the decisions of participants who had sent a deceptive message in round 1 (85% of the participants). Then – to obtain a measure of escalation – we calculated the difference between the message scores (which could run from 1 to 10) of round 2 minus those of round 1^8^. As a result a difference score of 0 shows escalation by engaging in a new unethical act of deception that is as unethical as the deceptive behavior one performed in round 1 (the same level of deception in round 2 than in round 1), while positive scores denote escalation by engaging in a new unethical act at a higher level of intensity (more extreme deception in round 2 than in round 1).

The escalation scores were distributed in a non-normal way: 68% of the participants sent the same message in the first and in the second round, resulting in an escalation score of “0.” Also, there were infrequent extreme deviations from the mean (i.e., scores of −7, −5, and 4). To test whether indeed the distribution was non-normal, the kurtosis was calculated, and indeed appeared to be extremely high (z-score = 12.61), suggesting that a transformation of the escalation score was warranted (see Field, 2005). We therefore transformed the escalation measure into a trichotomous one: participants with lower levels of deception in round 2 than in round 1 were coded as “−1” (de-escalation), and those with higher levels of deception in round 2 than in round 1 one as “1” (escalation at an increased intensity level). Those with the same levels of deception in round 2 and 2 were coded as “0” (escalation at the same intensity level). This led to a kurtosis within the normal range (z-score = 0.29).

**FIGURE 3:**
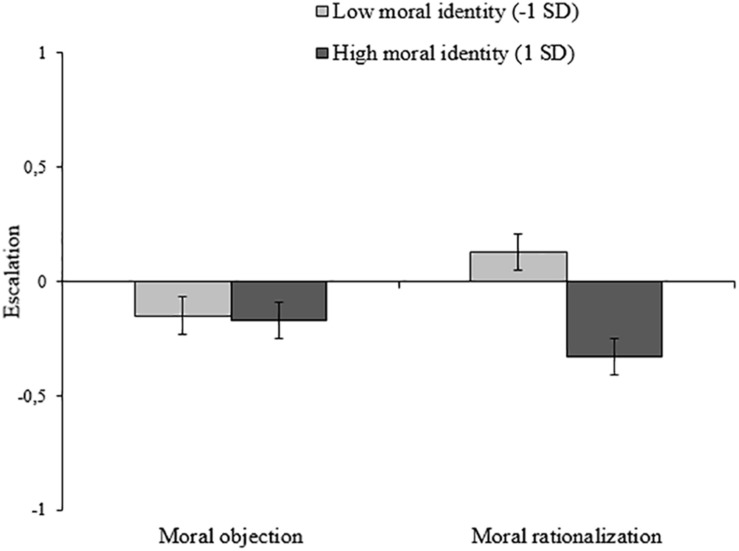
Escalation of unethical behavior (lying) as a function of moral argumentation and moral identity (Study 3).

To test whether moral identity modulated the tendency to escalate, three regression steps were performed. Moral identity was standardized and cross products were calculated for the interaction terms. First, the transformed escalation score was regressed on the moral argumentation and on moral identity. This model was marginally significant *F*(2,90) = 2.53, *p* = 0.09, *R*^2^ = 0.05) and rendered a main effect for moral identity (*B* = −0.12, *p* = 0.03). There was no main effect of moral rationalization (*B* = 0.03, *p* = 0.62), again indicating that moral rationalization, overall, had no influence on escalation. However, when the two-way interaction between moral identity and the moral argumentation was included, the model changed significantly, Δ*F*(1,89) = 4.26, *p* = 0.04, *R*^2^ = 0.10, Δ*R*^2^ = 0.04, and the Moral Argumentation × Moral Identity interaction was significant (*B* = −0.11, *p* = 0.04). Follow-up analyses of this interaction showed that, albeit marginally, moral rationalization (as compared to moral objection) increased escalation among low moral identifiers (*B* = 0.14, *p* = 0.07) and did not increase escalation among high moral identifiers (*B* = −0.08, *p* = 0.31). See Figure 3^9^.

### Discussion

Study 3 confirms that moral rationalization contributes more to the escalation of unethical behavior when moral identity is low than when it is high. Importantly, we were able to reveal this process by using real behavioral choices. In contrast to studies 1 and 2, participants were not asked to imagine having engaged in morally questionable behavior, but they actually did engage in such behaviors. Moreover, by comparing behavior in round 1 and 2, we were able to show the direction of the change. While for about two-thirds (68%) of the participants escalation took the form of showing new unethical behavior at the same level of intensity as before, the remaining participants did change the intensity. Our analysis also shows the direction of change. The interaction we observed suggests that escalation in levels of intensity tended to be more likely among low moral identifiers than among high moral identifiers, when they had engaged in moral rationalization.

## General Discussion

Moral rationalizations are often thought to facilitate the continuation, or even escalation, of immoral behavior. However, this causal relation was never put to the test. Most of the literature on escalation of immoral behaviors consists of theory papers (e.g., Ashforth and Anand, 2003; Tenbrunsel and Messick, 2004; Anand et al., 2005; Zyglidopoulos et al., 2009). A few exceptions are empirical research about the role of commitment in escalation of killing (Martens et al., 2007, 2010), about gradual (and unnoticed) erosion of unethical practices (Gino and Bazerman, 2009), and about the relation between moral disengagement and escalation (Welsh et al., 2015), showing mainly the relationship between one’s propensity to morally disengage (as an individual difference variable) and (un)ethical behavior (Bandura et al., 1996; Detert et al., 2008; Moore et al., 2008). Only few studies tapped into causal influences of moral rationalization, but did so by testing moral rationalization effects in indirect ways. They showed that situations that allow for moral rationalizations, for example, another person benefiting from the unethical act (Wiltermuth, 2011), circumstances decreasing one’s sense of responsibility (Bersoff, 1999), or the presence of counterfactual information about “how circumstances could have been different” (Shalvi et al., 2011), evoke more unethical behavior. Whether the actual generation of moral rationalizations induces immoral behavior (and, more specific, the escalation of it) was not yet established.

By explicitly asking participants to engage in moral argumentation, we were able to identify a causal path. Across the three studies we showed that generating moral rationalizations for (as opposed to moral objections to) immoral behaviors evokes further immoral behaviors more among those with a low moral identity than among those with a high moral identity. Importantly, these effects emerged primarily after having engaged in immoral acts. This suggests that moral rationalization may particular be an issue of concern when it serves low moral identifiers to justify one’s previous unethical behavior, as it then opens the path for engaging in more unethical behavior. Further, our studies suggest that effects of *post hoc* rationalizations may show themselves in new unethical behaviors that resemble the past transgressions in terms of unethicality, but also in self-chosen increased levels of unethicality (as shown in Study 3 where low moral identifiers were more likely to choose a more extreme form of dishonesty after having engaged in moral rationalization).

The fact that the effects *post hoc* rationalizations can show themselves in different ways, also raises the question of whether escalation of unethicality may also occur over different domains of behavior. For example, does *post hoc* rationalizations of a specific behavior performed earlier (e.g., excessive reimbursing), also promote future rule breaking in other domains (e.g., cheating on an exam)? Previous theorizing on rationalizations suggests that this can be the case. More precisely, it has been argued that moral rationalizations may be excessive in relation to the actual act, which provides an impetus for other types of immoral acts (Zyglidopoulos et al., 2009). For example, after having engaged in excessive reimbursing a person may rationalize that “everyone is dishonest now and then.” Such rationalization would also cover cheating on an exam. However, the rationalization “my reimbursement is only very small considering the revenue of the organization” will not automatically form a rationalization for cheating on an exam. Hence, whether engaging in moral rationalizations fosters escalation of unethical behaviors in different domains, probably depends on the type of moral rationalization that people engage in. This would be an interesting topic for further research.

It should be noted that in our studies, we always contrasted the generation of moral rationalizations with that of generating moral objections. Because both types of moral argumentation require one to consider the moral connotations of the behavior, this allowed us to test whether the nature of moral argumentations affects subsequent decisions. This also means that we did not include a control condition in which participants, for example, would not engage in any moral argumentation (i.e., a setting in which the morality of the behavior would not be additionally cued by moral argumentation). Future research could include such a condition, which would allow for studying whether the salience of morality concerns would have an additional impact.

By studying how moral argumentations about prior behaviors affected subsequent behavior we zoomed in on *post hoc* moral rationalizations, which should be distinguished from moral rationalizations that people may use to facilitate behavior that one is about to show (*ex ante*). *Post hoc* moral rationalization refers to the aftermath of unethical behavior; a highly relevant phase considering that we all behave somewhat unethical once in a while. As this forms a threat to the self-image of being moral and honest (Mazar et al., 2008), people somehow need to deal with this. Justifying why the previous unethical behavior was not so unethical may seem an effective way to cope with the feelings of guilt as a result of that behavior, thereby maintaining their moral self-esteem. The relevance of the current studies, of course, is that we demonstrated that its impact is not restricted to dealing with the past; its effects may extend to future behaviors as well. The current findings revealed this connection especially for those with a low moral identity. An interpretation of this finding is that *post hoc* moral rationalization was less likely to convince those with a high moral identity that the unethical behavior is morally acceptable, and hence less successful in reducing guilt. Hence, engaging in *post hoc* moral rationalizations may especially induce low identifiers to tread the path of unethical escalation.

By inducing participants to engage in moral argumentation, we were able to further illuminate its causal path, and study to what extent the path taken is dependent on one’s moral identity. However, one may raise the questions of how the results relate to settings in which the process of generating argumentations is not externally stimulated and to what extent high moral identifiers spontaneously engage in moral rationalizations after having shown unethical behavior. To our knowledge, this issue has not yet been addressed in the literature. In any case, the studies that have been conducted would not support the notion that high moral identifiers never engage in moral rationalizations. True, there is previous research that finds that moral identity negatively correlates with the one’s propensity to morally disengage. However, these correlations are not critically large, and vary from −0.24 (Detert et al., 2008), and −0.27 (Vitell et al., 2011), to −0.42 and −0.55 (Moore et al., 2012). This suggests that there is a significant group of high moral identifiers with a high propensity to morally disengage. Moreover, even high moral identifiers with a *low* propensity to morally disengage might engage in small unethical acts and justify this with *post hoc* moral rationalization. After all, those with a high moral identity might be especially motivated to restore their moral self-image after doing so (see also Mulder and Aquino, 2013). Some support for this was indeed found by Aquino and Becker (2005) who tested the relation between self-perceived moral attributes and neutralization techniques in the context of negotiation. They found that, after concealing information in a negotiation, self-perceived moral attributes were positively related to certain types of neutralization strategies. Future research could focus on the question how moral identity and engaging in moral rationalization relate, and take the distinction between *post hoc* and *ex ante* moral rationalization into account.

While the main focus in our theorizing has been on the role of moral rationalizations, the effect of generating moral objections on behavior is also interesting. The data of Study 1 suggest that after having behaved ethically, generating moral objections for unethical behaviors makes high moral identifiers behave more ethically. Possibly, generating (*post hoc*) moral objections may work as an extra confirmation of one’s previous ethicality. This might have been more impactful for high moral identifiers as they are the ones who especially value being a moral person. For them, such a confirmation might work as an extra motivating power to act in line with what they previously did, or to express more strong opinions in line their previous ethical behavior. However, since this effect was unexpected and inconsistent (the effect was only found in Study 1), it requires future research to further address how the effects of moral objections be modulated by moral identity.

A limitation of our research concerns the identification of the psychological process that underlies our behavioral findings. We suggested that, when moral identity is high, engaging in moral rationalizations is less successful in convincing people that their previous immoral act is morally acceptable, and thus is also less successful in alleviating them from guilt. We did measure guilt and moral acceptability perceptions. The results for these measures are presented in the supplementary material^10^. Indeed, the data of Study 2 show support for the notion that the differential effect of MR on subsequent immoral behavior can be explained by moral acceptability perceptions and guilt. However, the data of Studies 1 and 3 do not. This could be due to the fact that in these studies, the measure of moral acceptability was taken toward the end of the study, and the measure of guilt was measured after, rather than before, subsequent behavior. Nevertheless, this explanation is *post hoc* and thus we should be careful with drawing definite conclusions from this. Future research is required to determine to what extent the escalatory effect of moral rationalizations (and how this stronger for low moral identifiers) is explained by moral acceptability perceptions and guilt.

While the current findings suggest that *post hoc* moralizations and moral identity impact how people deal with unethical behavior, we do not want to overstate our case by claiming that these factors alone suffice to explain the ethical decisions people make. Indeed, we take to position that – like most of the decisions people make – behavior is multi-determined. Unethical decision making may also be related to other individual differences, and situational features we did not investigate here. The relatively low levels of explained variance that could be traced back to *post hoc* rationalizations and moral identity serve as a reminder that we should not overstate their explanatory power. The findings do show, however, that it may be worthwhile to further explore their effects.

## Conclusion

Everyone behaves unethically once in a while. This paper tested conditions under which such unethical acts either remain single occasions or are continued. The results show that engaging in *post hoc* moral rationalizations may especially lead low moral identifiers engage in new unethical acts a second time, and even increase the intensity level of unethicality. High moral identifiers, are less likely to follow this path. With these results, the current paper contributes to the understanding of continuation and escalation of unethical behavior. It is our hope that the current paper will inspire and help both scientists and practitioners to identify the conditions that determine whether inevitable occasions of unethical behavior go from bad to worse or remain single occasions.

## Data Availability Statement

All datasets generated for this study are available at https://osf.io/rkgcs/files/.

## Ethics Statement

The studies involving human participants were reviewed and approved by the Behavioural Research Lab Ethics Committee of the Faculty of Economics and Business, University of Groningen (Studies 1 and 3) or were in line with the ethics policies of the Faculty of Social Sciences, Tilburg University (Study 2). The participants provided their written informed consent to participate in the studies.

## Author Contributions

LM developed the theoretical basis of the manuscript, designed the studies, collected and analyzed the data, and wrote the manuscript. ED contributed to the theoretical basis and writing of the final manuscript.

## Conflict of Interest

The authors declare that the research was conducted in the absence of any commercial or financial relationships that could be construed as a potential conflict of interest.

## Footnotes

1 For exploratory reasons we also varied the size of this bill, being either €20 or €100. This did not affect the results in any way, so these two versions were aggregated. Controlling for this variable rendered similar results.

2 We also included several exploratory measures: power, need for consistency, motivation to compensate, emotions (among which guilt), some checks, rule breaking and deterrence perceptions, moral acceptability, and a moral rationalization Likert scale. Analyses on moral acceptability perceptions and guilt are presented in the supplementary material.

3 A critical reader could argue that our results may have been due to the fact that it is harder for high moral identifiers to generate compelling moral rationalizations, which may have made the moral rationalizations of high moral identifiers less influential than those of low moral identifiers. To check this, the pleas that respondents wrote were coded on the length (number of words) and “compellingness” (by two raters on a Likert scale, Pearson’s *r* = 0.69). Analyses showed that length and compellingness of the plea were not influenced by an interaction of moral identity and moral argumentation. Also, controlling for length and compellingness of the plea did not change the results.

4 We also checked this by asking participants at the end of Study 2 whether they had ever experienced a situation in which they had been offered too much change after buying something; 75% of the participants noted that they indeed had such an experience. Of these participants, 10% said they had not mentioned it and kept the money, 23% had mentioned it and returned the money; the remaining 67% reported having shown both behaviors: they had at least once kept the money and at least once returned the money. These numbers showed that the setting of receiving too much change is indeed familiar to most, and that it does create some ambiguity on how to respond.

5 We also included several exploratory measures: perspective taking, social value orientation, emotions (among which guilt), a moral rationalization Likert scale, moral acceptability perceptions and realism checks. Analyses on moral acceptability perceptions and guilt are presented in the supplementary material.

6 For the same reason as in Study 1, length and compellingness of the pleas were coded by two independent raters (Pearson’s *r* = 0.80). These measures were not influenced by a combination of moral identity and moral argumentation had no interactive effect on these two measures. Also, controlling for these two measure did not change the results.

7 We also included several exploratory measures: a few personality measures, emotions, commitment, perceptions of both decisions, choice strategy, a moral rationalization Likert scale, moral acceptability, self-ratings, intentions to donate to charity, and some checks. Analyses on moral acceptability perceptions and guilt are presented in the supplementary material.

8 Because for this analysis we excluded those who had sent a truthful message in round 1, these scores ranged from 2 to 10.

9 As in Studies 1 and 2, length and compellingness of the pleas were coded, now by three independent raters [*ICC* (2,3) = 0.77]. Moral identity and moral argumentation had no combined effect on these two measures. Also, controlling for these two measure did not change the results.

10 https://osf.io/rkgcs/files/

## Appendix: Options of the Cheating Game (Study 3)

### Options and Their Distributions

Option A: you get €0, player 2 gets €50

Option B: you get €5, player 2 gets €45

Option C: you get €10, player 2 gets €40

Option D: you get €15, player 2 gets €35

Option E: you get €20, player 2 gets €30

Option F: you get €30, player 2 gets €20

Option G: you get €35, player 2 gets €15

Option H: you get €40, player 2 gets €10

Option I: you get €45, player 2 gets €5

Option J: you get €50, player 2 gets €0.
